# Microarray analysis of Foxl2 mediated gene regulation in the mouse ovary derived KK1 granulosa cell line: Over-expression of Foxl2 leads to activation of the gonadotropin releasing hormone receptor gene promoter

**DOI:** 10.1186/1757-2215-3-4

**Published:** 2010-02-18

**Authors:** Jean M Escudero, Jodi L Haller, Colin M Clay, Kenneth W Escudero

**Affiliations:** 1 Department of Biological and Health Sciences, Texas A&M University- Kingsville, Kingsville, TX, USA; 2 Biomedical Imaging and Bioengineering, National Institutes of Health, Bethesda, MD, USA; 3 Department of Biomedical Sciences, Colorado State University, Fort Collins, CO, USA

## Abstract

**Background:**

The Foxl2 transcription factor is required for ovarian function during follicular development. The mechanism of Foxl2 regulation of this process has not been elucidated. Our approach to begin to understand Foxl2 function is through the identification of Foxl2 regulated genes in the ovary.

**Methods:**

Transiently transfected KK1 mouse granulosa cells were used to identify genes that are potentially regulated by Foxl2. KK1 cells were transfected in three groups (mock, activated, and repressed) and twenty-four hours later RNA was isolated and submitted for *Affymetrix *microarray analysis. *Genesifter *software was used to carry out analysis of microarray data. One identified target, the gonadotropin releasing hormone receptor (GnRHR) gene, was chosen for further study and validation of Foxl2 responsiveness. Transient transfection analyses were carried out to study the effect of Foxl2 over-expression on GnRHR gene promoter-luciferase fusion activity. Data generated was analyzed with *GraphPad Prism *software.

**Results:**

Microarray analysis identified 996 genes of known function that are potentially regulated by Foxl2 in mouse KK1 granulosa cells. The steroidogenic acute regulatory protein (StAR) gene that has been identified as Foxl2 responsive by others was identified in this study also, thereby supporting the effectiveness of our strategy. The GnRHR gene was chosen for further study because it is known to be expressed in the ovary and the results of previous work has indicated that Foxl2 may regulate GnRHR gene expression. Cellular levels of Foxl2 were increased via transient co-transfection of KK1 cells using a Foxl2 expression vector and a GnRHR promoter-luciferase fusion reporter vector. The results of these analyses indicate that over-expression of Foxl2 resulted in a significant increase in GnRHR promoter activity. Therefore, these transfection data validate the microarray data which suggest that Foxl2 regulates GnRHR and demonstrate that Foxl2 acts as an activator of the GnRHR gene.

**Conclusions:**

Potential Foxl2 regulated ovarian genes have been identified through microarray analysis and comparison of these data to other microarray studies. The Foxl2 responsiveness of the GnRHR gene has been validated and provided evidence of Foxl2 transcriptional activation of the GnRHR gene promoter in the mouse ovary derived KK1 granulosa cell line.

## Background

The transcription factor Foxl2 is vital to ovarian function as evidenced by the identification of mutations in the gene encoding FoxL2 that result in the condition known as blepharophimosis/ptosis/epicanthus inversus syndrome (BPES) and in some cases, premature ovarian failure (POF) [1]. Type I BPES is characterized by eyelid malformation and POF suggesting that the expression of FoxL2 is critical in the developing eyelid as well as in the maintenance of ovarian function. Foxl2 has also been implicated in the process of sex determination in mice [2] as well as in humans [3].

Foxl2 knockout studies in the mouse provide compelling evidence of the critical role that Foxl2 plays in ovarian function. In a study in which a portion of the Foxl2 coding region (amino acids N-61) was fused to the β-galactosidase gene (*LacZ*), homozygous Foxl2-lacZ mice exhibited ovarian failure resulting from the absence of granulosa cell differentiation at an early stage of follicular development [4]. These investigators observed that follicles were activated and underwent apoptosis, leading to progressive follicular depletion and ovarian atresia. A second study in which both copies of Foxl2 were completely knocked out determined that the development of granulosa cells was blocked at the point of primordial follicle formation [5].

In order to understand the mechanism through which Foxl2 functions in follicular development, ovarian specific target genes must be identified. Microarray analysis has been performed previously by other groups using strategies that differ from this present study. The first study involved over-expression of FoxL2 in the human KGN granulosa cell line and *Nimblegen *gene chips [6]. A more recent study used Foxl2 knockout mice and whole ovary preparations of RNA for their analyses. Both *Affymetrix *and Agilent gene chips were used by these investigators [7].

In this present study, mouse Foxl2 target genes were identified using *Affymetrix *microarray analysis. The effect of Foxl2 mediated gene regulation was examined through the use of vectors expressing Foxl2 fusion proteins designed to either activate or repress gene expression (fusions are described in Methods section below). The KK1 mouse granulosa cell line is an excellent system for these studies in that these cells exhibit granulosa cell characteristics such as gonadotropin responsiveness and inhibin expression [8]. In addition, KK1 cells are easy to maintain in culture and can be transfected with high efficiency. The data generated using the *Affymetrix *microarray analysis have been compared to those found in the above mentioned studies and many of the target genes identified are common to those studies [6,7].

In addition, a candidate gene identified in the microarray analysis was chosen for further study. The GnRHR gene was chosen based on the results of our previous study which implicated Foxl2 in the regulation of GnRHR in the pituitary derived αT3-1 cell line [9]. This present study provides the first evidence that Foxl2 affects the expression of GnRHR in the ovary. Foxl2 regulation of the GnRHR gene has been examined in KK1 granulosa cells through the use of transient co-transfection studies of a GnRHR promoter-luciferase fusion construct and a wild type Foxl2 expression vector. The results of these analyses suggest that Foxl2 is a positive regulator of the murine GnRHR gene promoter.

## Methods

### Cell culture

The KK1 granulosa cell line was a gift from Dr. Ilpo Huhtaniemi whose laboratory developed the cell line and from Dr. Deborah Segaloff who sent us the cell line. The cells are grown in DMEM/F12 (50/50) containing 10% heat inactivated FBS (Gemini Bioproducts; West Sacramento, CA), 100 μg/ml penicillin-streptomycin, and 0.25 μg/ml amphotericin B. All cell culture reagents other than FBS were purchased from Mediatech; Manassas, VA.

### *Affymetrix *microarray analysis

The KK1 granulosa cell line was chosen as a model system for this study as a result of numerous published studies as well as our own observations suggesting that the site of action of Foxl2 during follicular development is the granulosa cell. However, due to endogenous expression of Foxl2 in KK1 cells, simple over-expression resulting from transfection of a Foxl2 expression vector may not affect levels of gene expression sufficiently to be detected efficiently by microarray analysis. Therefore, in order to increase the potential of Foxl2 to alter gene expression levels of putative target genes, two fusions were constructed consisting of Foxl2 fused to the activation domain of the *Herpes simplex *virus VP16 transcription factor (Foxl2-VP16) and Foxl2 fused to the repression domain of the *murine *MAD transcription factor (Foxl2-MAD). Levels of gene expression were compared between mock transfected cells and those that were transfected with Foxl2-VP16 and Foxl2-MAD, respectively.

#### Foxl2 fusion protein derivatives

The construction of Foxl2-VP16 was described previously and was shown to function as a specific activator of transcription [9]. Foxl2-MAD consists of the 40 amino acid N-terminal mSin3 interaction domain (SID) of the Mad transcription factor fused to Foxl2 in the expression vector pcDNA 3.1 (Invitrogen Corporation; Carlsbad, CA). The repression activity of the Mad-SID is mediated through interactions of the Mad N-terminus with the mammalian Sin3 co-repressor protein [10,11]. The effectiveness of the Sin3 binding domain of Mad in silencing gene expression when fused to DNA binding proteins has been demonstrated in two studies. One group fused the Mad SID to the *c-Jun *DNA binding domain and demonstrated specific inhibition of transcription mediated by binding to *AP-1 *binding site elements [12]. Another group of investigators fused Mad SID to the tetracycline receptor and demonstrated tetracycline mediated gene repression via binding to the tetracycline operator sequence [11]. The ability of Foxl2-Mad and Foxl2-VP16 fusion constructs was tested utilizing the Foxl2-VP16 responsive luciferase reporter 3X-GRAS-Luc [9]. In transient transfections of KK1 cells, Foxl2-VP16 resulted in a greater than 50-fold increase in luciferase expression. The co-transfection of Foxl2-Mad attenuated the ability of Foxl2-VP16 to activate luciferase expression by 95% (data not shown). Therefore, these fusion vectors are appropriate for activation and repression studies of Foxl2 specific genes.

#### Microarray transfection

KK1 cells were grown in 150 mm tissue culture plates to 50% confluence. Three plates were mock transfected with 30 μl of Fugene 6 transfection reagent (Roche Applied Science; Indianapolis, IN) and 10 μg of empty vector (pcDNA 3.1). Three plates were transfected with 10 μg of Foxl2-VP16 expression vector in 30 μl Fugene reagent and 3 plates were transfected with 10 μg of Foxl2-MAD expression vector in 30 μl Fugene reagent. After 24 hours incubation at 37°C the cells were harvested and polyA^+ ^RNA was isolated using RNeasy (Qiagen Inc.; Valencia, CA) according to manufacturer's procedures. Ten μg of RNA isolated from each plate were diluted to a concentration of 1 μg/μl and a total of 9 samples were submitted to the Colorado State University *Affymetrix *core facility for analysis.

#### Microarray and data analysis

Nine *Affymetrix *mouse genome 430 2.0 chips were used (one for each plate of KK1 cells). Our microarray data can be downloaded from the web site, http://www.ncbi.nlm.nih.gov/geo, via the accession number GSE18891. Data obtained from the chips was analyzed using the *Genesifter *Program (VizX Labs LLC; Seattle, WA).

Two independent analyses were performed. In the first, our data was analyzed using pairwise analysis, mean normalization, and T-test statistical analysis (P < 0.05). In the second, we compared our data to that of Garcia-Ortiz et al. [7]. Eighteen *Affymetrix *CEL files representing the developmental stage embryonic days 13 and 16, and birth were downloaded from http://www.ncbi.nlm.nih.gov/geo, via the accession number GSE12989 [7]. These files were uploaded along with our 9 *Affymetrix *CEL files together into *Genesifter *for analysis using the MAS5 advanced upload method. A project analysis in was set up in *Genesifter *with the following parameters: the cutoff threshold was set at 1.8 fold with T-test statistical analysis (mouse) or anova (KK1), and P < 0.05.

### Transient co-transfection and luciferase assays

#### Expression vectors

The original GnRHR promoter luciferase fusion reporter vector (pMGR-600 Luc) was described previously [13]. In this study, the plasmid -600 Luc was created by subcloning the 600 base pair fragment of the mouse GnRHR promoter into the expression vector pGL3 basic (Promega Corporation; Madison, WI). The Foxl2 expression vector (pFoxl2) consists of the mouse Foxl2 open reading frame inserted in pCDNA 3.1 (Invitrogen Corporation; Carlsbad, CA). The renilla luciferase control vector phRL-CMV is part of the dual luciferase assay system (Promega Corporation; Madison, WI).

#### Transfection

KK1 cells were plated at a density of 2.5 × 10^5 ^cells per well of 24 well tissue culture plates the day the transfections were carried out. Transfections included 0.3 μg -600 Luc reporter plasmid, 3 ng of phRLCMV renilla luciferase vector to normalize for transfection efficiency, and either 0.3 μg pcDNA 3.1 (as a control) or 0.3 μg pFoxl2. DNA was mixed with DMEM/F12 medium containing Fugene 6 reagent (Roche Applied Science; Indianapolis, IN) at a ratio of 4:1 Fugene to total DNA and added to cells.

#### Luciferase assays

Transfected cells were incubated for 24 hours at 37°C and washed 3 times with phosphate buffered saline. Cells were lysed and the *Dual-luciferase *assay was performed according to manufacturer's instructions (Promega Corporation; Madison, WI) using a 20/20^**n **^luminometer (Turner Biosystems; Sunnyvale, CA). Each transfection was carried out in triplicate and experiments were carried out a total of 4 times. Data was analyzed with the paired T-test using *GraphPad Prism *software (GraphPad Software, Inc.; La Jolla, CA).

## Results

### *Affymetrix *microarray analysis

In order to increase the effectiveness of this analysis, Foxl2 derivatives consisting of the entire open reading frame of Foxl2 fused to either the strong transcriptional activation domain of *Herpes simplex *virus VP16 [9] or the Mad protein-SID domain, a repressor of transcription [10,14] were used. These Foxl2 fusion proteins can be used as tools for gene discovery as the DNA binding domain of Foxl2 will guide either the activation or repression domain to the promoter regions of genes that are normally regulated by Foxl2 via specific DNA binding. Theoretically, Foxl2-VP16 should stimulate genes that are normally regulated by Foxl2, particularly those that are normally repressed in KK1 cells. Conversely, Foxl2-MAD should function to repress genes normally regulated by Foxl2, especially those that are normally active in KK1 cells. In this study, levels of gene expression in murine KK1 granulosa cells transfected with the fusion protein expression vectors were compared to mock transfected cells. A pairwise analysis was performed with the following groupings: mock vs. VP16 transfected cells (group NA); mock vs. Mad transfected cells (group NR); and Mad vs. VP16 transfected cells (group RA). The cumulative listings of genes for these groups are found in Additional Files 1, 2, and 3 respectively. The results of the pairwise analysis are summarized in the comprehensive listing of 636 potential targets of the Foxl2 transcription factor (Additional File 4). Only genes of known function are listed and all exhibited at least a 1.75 fold change in expression levels.

In order to validate these findings, we carried out a comparison of our data to data derived from two other microarray studies [6,7]. One group used the human KGN granulosa cell line and over-expression of FoxL2 to identify potential targets and qPCR to confirm numerous FoxL2 regulated genes [6]. A second group used mouse Foxl2 knockouts and whole ovarian preparations for microarray studies as well as analysis of other investigator's microarray data [7].

Garcia-Ortiz et al. [7] used *Affymetrix *gene chips identical to those used in our study to compare developmental stage specific gene expression levels in ovary preparations from wild type and Foxl2 knockout mice at embryonic day 13 (E13), embryonic day 16 (E16), and birth (P0). This allowed us to compare our raw data directly to theirs and determine the similarity between differentially expressed genes in our KK1 study and their stage specific study. In this analysis, we compared differentially expressed genes from mock, VP16, and Mad transfected KK1 cell groups combined (Additional file 5) to mouse samples [7] that compared wild type to Foxl2 knockout for stages E13 (Additional file 6); E16 (Additional file 7), and P0 (Additional file 8).

**Figure 1:**
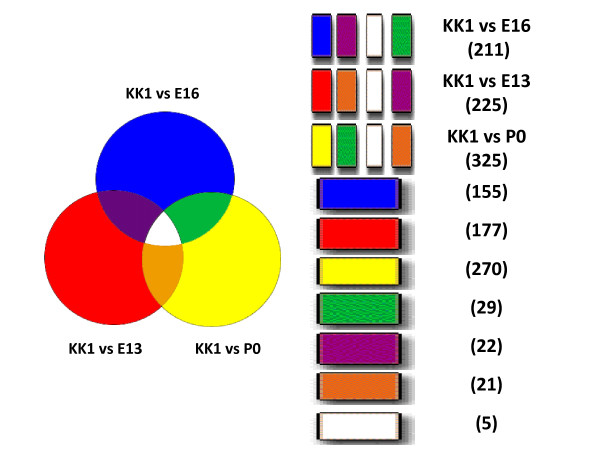
**Comparison of differential gene expression in KK1 cells to mouse ovary developmental stages**. *Genesifter *software was used to determine differentially expressed genes in KK1 cells and mouse *in vivo *samples [7]. The KK1 analysis for differentially expressed genes generated a cumulative listing of 2520 differentially expressed genes among the mock, VP16, and mad transfected cell groups combined (Additional file 5). The mouse samples compared wild type to knockout for each stage as follows: E13, 3289 genes (Additional file 6); E16, 2995 genes (Additional file 7); and P0, 4330 genes (Additional file 8). Comparisons between groups of genes were performed with *Genesifter Intersector*. The comparisons are represented by circles and are as follows: KK1 vs. E16; KK1 vs. E13; and KK1 vs. P0. The number of genes in each grouping corresponding to color codes is indicated on the right. A listing of all genes may be found in Additional file 9.

The "*Intersector" *subroutine in *Genesifter *allowed us to find commonalities between the groups of genes in Additional files 5, 6, 7, 8. The comparisons are the following: KK1 vs. E16, KK1 vs. E13, and KK1 vs. P0 (Figure 1 and Additional file 9). This analysis resulted in the discovery of 360 new genes of known function that were shared between the KK1 and mouse ovary studies increasing the total number of potential Foxl2 target genes to 996 (Additional file 4).

We then turned our attention to the human KGN cell line study of Batista et al. [6]. In comparing their confirmed human gene list to our mouse gene list, 3 common genes were identified. These were *Mrgpre*, *Maff*, and *Rspo3 *(Additional file 10). Comparison of their comprehensive listing with our study's list of genes resulted in finding a total of 42 genes common to both (Additional file 10).

In order to begin to understand the significance of the genes common to the three microarray studies in the ovary, we then searched the *Ovarian Kaleidoscope Database (OKdb) *to determine if other investigators had published evidence of ovarian expression of these genes. A total of 71 of the genes were found to be expressed in the ovary and have been categorized according to function: Gene Regulation (Table 1), Signaling (Table 2), and Metabolism, Cell Adhesion, Cytoskeletal, and Structural (Table 3).

**Table 1:** Gene Regulation

Gene Symbol	Name	Entrez Gene ID	Group
Bcl11a**	B cell CLL/lymphoma 11a (zinc finger protein)	14025	MA -4.5
Bub3	Budding uninhibited by benzimidazoles 3 homolog	12237	E16
Cdc25a	Cell division cycle 25 homolog A (S. pombe)	12530	P0
Dazl	deleted in azoospermia-like	13164	MA -3.1
Etv1	Ets variant gene 1	14009	RA+6.5, E16
Gabpa	GA repeat binding protein, alpha	14390	P0
Greb1	Growth regulation by estrogen in breast cancer 1	268527	E13, E16
Hoxb5**	Homeo box B5	15413	RA-5.1, E16
Id4	Inhibitor of DNA binding 4	15904	MA -5.6
Mcm7	Minichromosome maintenance deficient 7	17220	E16, P0
Nr4a2	Nuclear receptor subfamily 4, group A, member 2	18227	E16
Plagl1	pleiomorphic adenoma gene-like 1	22634	MA +5.3
Serpine2	serine (or cysteine) peptidase inhibitor, clade E, member 2	20720	MR +2.4
Snw1	SNW domain containing 1	66354	E16, P0
Sox21*	SRY-box containing gene 21	223227	MR +2.5
Zfp106	zinc finger protein 106	20402	MR +5.1

All genes listed that do not have an asterisk after the gene symbol are common to our study and the *in vivo *in mouse ovary study only [7]. Genes denoted by a single asterisk* are common to our study and the human KGN cell line only [6]. Those indicated by ** are common to all three studies. The (-) symbol indicates fold decreased expression. The (+) symbol indicates fold increased expression. MA compares mock (M) transfected cells to activated (A) cells (Foxl2-VP16 transfected). MR compares mock (M) transfected to repressed (R) cells (Foxl2-Mad transfected). RA compares repressed to activated cells. E16 and E13 are mouse embryonic stages day 16 and 13, respectively. P0 is mouse birth stage.

**Table 2:** Signaling

Gene Symbol	Name	Entrez Gene ID	Group
Akt1	Thymoma viral proto-oncogene 1	11651	E16
Akt2	Thymoma viral proto-oncogene 2	11652	P0
Ccr2	chemokine (C-C motif) receptor 2	12772	RA +4
Ctla4**	cytotoxic T-lymphocyte-associated protein 4	12477	RA -5.2
Dlg5	Discs, large homolog 5	71228	MA -3.7, RA -3.6
Eda**	ectodysplasin-A	13607	RA -3.9
Fgf8	fibroblast growth factor 8	14179	MR -4.8
Gnrhr***	Gonadotropin releasing hormone receptor	14715	MA -2.5, MR -3.3
Gucy1b3	Guanylate cyclase 1, soluble, beta 3	54195	MR -2.7
Irak1	Interleukin-1 receptor associated kinase 1	16179	E16
Ltk	Leukocyte tyrosine kinase	17005	MA -2.9
Mrgpre**	MAS-related GPR, member E	244238	NA-1.91
Npy1r*	Neuropeptide Y receptor Y1	18166	RA-1.79
Pard3	Par-3(partitioning defective 3)homolog(C. elegans)	263803	E13
Ppp1r1b	Protein phosphatase1, regulatory(inhibitor)subunit 1	19049	P0
Prlr	Prolactin receptor	19116	MA -7.3, MR -7.3
Ptpn6**	Protein tyrosine phosphatase, nonreceptor type 6	15170	MR-2.5, RA+2.1
Reln*	Reelin	19699	MR +7.7
Slit2	Slit homolog 2 (drosophila)	20563	MA -6, MR -11.6
Stc1	Stanniocalcin 1	20855	P0
Stk3	Serine/threonine kinase 3(STE20 homolog, yeast)	56274	E16
Thbd*	Thrombomodulin	21824	NA-2.3, NR-2.5
Tmsb10	Thymosin, beta 10	19240	RA +3.4
Wnt9a	Wingless related MMTV integration site 9a	216795	P0

All genes listed that do not have an asterisk after the gene symbol are common to our study and the *in vivo *in mouse ovary study only [7]. Genes denoted by a single asterisk* are common to our study and the human KGN cell line only [6]. Those indicated by ** are common to all three studies. The (-) symbol indicates fold decreased expression. The (+) symbol indicates fold increased expression. MA compares mock (M) transfected cells to activated (A) cells (Foxl2-VP16 transfected). MR compares mock (M) transfected to repressed (R) cells (Foxl2-Mad transfected). RA compares repressed to activated cells. E16 and E13 are mouse embryonic stages day 16 and 13, respectively. P0 is mouse birth stage.

**Table 3:** Metabolism/Cell Adhesion/Cytoskeletal/Structural

Gene Symbol	Name	Entrez Gene ID	Group
Acta2	Actin, alpha 2, smooth muscle, aorta	11475	E16
Bpgm	2,3-bisphophoglycerate mutase	12183	E16, P0
Casq1	Calsequestrin 1	12372	RA +4.4
Dlg5	Discs, large homolog 5	71228	NA-3.7, RA-3.6, E13
Erp29	Endoplasmic reticulum protein 29	67397	E16, P0
Hspg2	Heparin sulfate proteoglycan 2	15530	E16, P0
Itih5	Inter-alpha (globulin) inhibitor H5	209378	E16
Klk13*	Kallikrein related-peptidase 13	626834	RA +3.3
Odc1	Ornithine decarboxylase, structural 1	18263	E16
Slc12a2	Solute carrier family 12, member 2	20496	E16, P0
StAR***	Steroidogenic acute regulatory protein	20845	MA+2.7, MR+3.9
Thbs2	Thrombospondin 2	21826	E13, E16
Usp9x	Ubiquitin specific peptidase 9, X chromosome	22284	E13, P0
Vldlr	Very low density lipoprotein receptor	22359	MR +2.1

All genes listed that do not have an asterisk after the gene symbol are common to our study and the *in vivo *in mouse ovary study only [7]. Genes denoted by a single asterisk* are common to our study and the human KGN cell line only [6]. Those indicated by ** are common to all three studies. The (-) symbol indicates fold decreased expression. The (+) symbol indicates fold increased expression. MA compares mock (M) transfected cells to activated (A) cells (Foxl2-VP16 transfected). MR compares mock (M) transfected to repressed (R) cells (Foxl2-Mad transfected). RA compares repressed to activated cells. E16 and E13 are mouse embryonic stages day 16 and 13, respectively. P0 is mouse birth stage.

Three of the genes that we have included in Table 2 were not found in the *OKdb*: *Mrgpre, Ctla4 *and *Eda*. These are potentially important due to the fact that they are common to all three studies. The *StAR *gene was not found in either of the other group's data sets but included in Table 3 because it has been shown to be a human Foxl2 target gene [15]. Our finding that the mouse *StAR *gene is a Foxl2 target validates the approach chosen for this study. The *GnRHR *gene was not found in either of the other group's data sets and is noted in Table 2 because further evidence of Foxl2 regulation of this gene is provided in the section that follows.

### Transient co-transfection and luciferase assays

**Figure 2:**
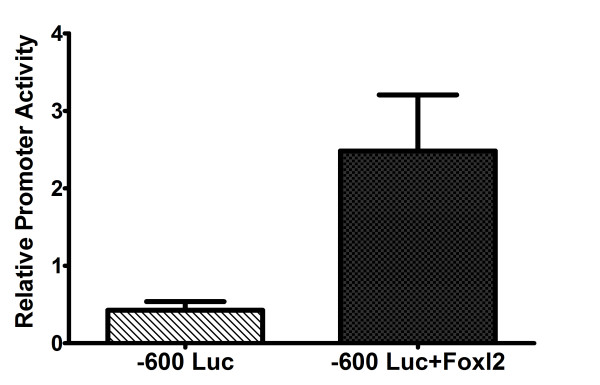
**Foxl2 over-expression causes activation of the GnRHR promoter**. The GnRHR promoter-firefly luciferase vector (-600 Luc) was co-transfected with either pFoxl2 or pcDNA 3.1. All transfections included the control vector phRLCMV that expresses renilla luciferase to correct for differences in transfection efficiency between samples. Firefly luciferase values were divided by renilla luciferase values to normalize for transfection efficiency. As an additional control, the promoter-less luciferase vector (pGL3 basic) was transfected with either the empty vector pCDNA3 or Foxl2 expression vector (pFoxl2) in order to show that Foxl2 did not affect the luciferase control vector (Data not shown). Data from four independent transfection experiments was combined to generate the graph in Figure 1. Each of the four experiments was performed in triplicate for a total of 12 data points represented in each column. Each of the experiments used different KK1 cell cultures and DNA preparations. Statistical analysis using *GraphPad Prism *software (paired T-test; p = 0.0067**) allowed us to determine that Foxl2 over-expression caused a 5.8 fold increase in promoter activity.

Transient co-transfection studies to determine the Foxl2 regulation of the GnRHr promoter were carried out using KK1 cells transfected with various combinations of luciferase reporter vectors and pcDNA 3.1 expression vectors. The effect of Foxl2 over-expression on the activity of the GnRHR promoter was determined by comparing the luciferase activity of the promoter in the presence of pFoxl2 (over-expression) to the activity of the promoter in the absence of Foxl2 over-expression (pcDNA 3.1). Foxl2 over-expression activated the GnRHR promoter 5.8 fold (Figure 2).

## Discussion

This study has resulted in an increased awareness of Foxl2 function in the ovary. First, through the use of microarray analysis we have added to the growing list of genes that appear to be regulated by Foxl2 and thus may play a role in follicular development in the ovary. Second, we have demonstrated the GnRHR gene promoter is regulated in a positive manner by the transcription factor Foxl2 in the KK1 granulosa cell line.

### *Affymetrix *microarray analysis

In an effort to increase the level of differential gene expression that could be induced by Foxl2 and thereby efficiently detected by microarray analysis, we have used Foxl2 derivatives in this study. This approach appears to have succeeded in that the mouse StAR gene was detected and had been previously demonstrated to be Foxl2 responsive in a human system [15]. Based on our experimental design, we would have predicted that in comparing mock to Foxl2-VP16 transfected cells (NA) all values would be positive due to VP16 transactivation. However, in looking through the genes beginning with the letter "A" in our comprehensive alphabetical listing (Additional file 4), 16 out of 22 in the NA category were negative. The simplest explanations for repression of gene expression by VP16 are provided by the authors of a study that also used a VP16 fusion for microarray analysis and also noted unexpected negative regulation [16]. These investigators speculated that VP16 caused the induction of repressors or squelching of coactivator activity [16].

The repressor induction mechanism for VP16 repression is a distinct possibility in light of recent studies that have explored the mechanisms involved in the control of Foxl2 transactivation activity. These investigators found that deacetylation of the Foxl2 protein by the SIRT1 deacetylase causes a decrease in Foxl2 transactivation [17]. Sirt1 was also identified as a Foxl2 regulated gene that is activated by Foxl2 [18]. In addition, these investigators demonstrated that the Foxl2 promoter is repressed by Sirt1 expression as part of a feedback mechanism of regulation in response to stress [18]. Therefore, Sirt1 induction could alter the activity of Foxl2-VP16, as well as repress other genes, resulting in down regulation of genes in Foxl2-VP16 transfected cells. A specific example is a study demonstrating that Sirt1 deacetylation of AP1 modulates its function and causes repression of the *Cox2 *gene [19].

The comparison of mock to Foxl2-Mad transfected cells (NR) of genes beginning with the letter "A" reveals 10 out of 12 are negatively regulated as expected (Additional file 4). The finding that only 2 out of the 12 were activated by Mad domain repression suggests that Foxl2-Mad functions more reliably as a repressor in comparison to Foxl2-VP16 as an activator in our study. Perhaps this is due to the Mad repression domain interacting specifically with the Sin3 complex deacetylase leading to chromatin remodeling [10]. On the other hand, the VP16 activation domain mechanism of transactivation is the result of interactions with a variety of factors including histone acetylases, basal transcription factors, and the coactivators CBP and Mediator to name a few [20,21]. Therefore, over-expression of VP16 fusion proteins may lead to repression due to competition for the factors needed for endogenous gene expression [22]. With this in mind, the use of an alternative activation domain with greater specificity in its interactions would have resulted in fewer false repression events. However, we should reiterate that this microarray study was intended to provide a listing of potential Foxl2 target genes and does not have the potential to discern Foxl2 regulatory mechanisms.

This study has compared microarray data from two *in vitro *studies that utilized the human KGN [6] and mouse KK1 (this study) cell lines respectively, and an *in vivo *study that used mouse Foxl2 knockouts [7]. The KK1 cell line was derived from a transgenic female mouse in which SV40 T antigen expression was driven by a 6 Kb inhibin alpha promoter fragment [8]. The mouse developed a large ovarian tumor that was collected after 5 months. The tumor cells had the morphological characteristics of granulosa cells. Subcultures were tested for their cAMP and steroidogenic response to chorionic gonadotropin and the culture with the strongest response (KK1) was characterized further. The KK1 cells were shown to be immortalized luteinizing granulosa cells that expressed LH and FSH receptors, steroidogenic enzymes, and inhibin alpha [8]. The KGN cell line was derived from a 73 year old woman in which granulosa cell carcinoma had recurred [23]. The KGN cells had steroidogenic activities similar to those of normal human granulosa cells and expressed functional FSH receptor [23]. Therefore, in comparisons of these studies, we would assume that the microarray data derived from the KGN and KK1 cell lines is representative of well differentiated granulosa cells while the mouse microarray data (E13, E16, and P0) represents less differentiated granulosa cells from embryonic stages and birth [7].

As seen in Figure 1, we do find evidence that this assumption is correct when we compare the number of genes shared between KK1 cells and the *in vivo *mouse data of Garcia-Ortiz et al. [7]. The number of shared genes increases from the embryonic stages (211&225 genes respectively) to 325 genes at birth (P0), indicating that KK1 cells have transcriptional profile more like that of a mature granulosa cell *in vivo*. Further similarities as well as differences in shared genes among the three comparison groups represented by the different colors in Figure 1 can be found in individual sheets in Additional file 9. Of the five genes that are shared by all groups (Figure 1-white), three have known functions: *Pa2g4, Rab28*, and *Thbs2. Pa2g4 *stands for proliferation-associated 2G4, a transcription factor involved in cell growth and signalling [24]. *Rab28 *is a *Ras *oncogene family member involved in the regulation of membrane trafficking [25]. *Thbs2 *is also found in Table 3, and encodes thrombospondin 2, an antiangiogenic protein involved in follicle development [26]. The two genes of unknown function are *RIKEN cDNA 5033428C03 *which encodes the hypothetical protein LOC74728 (*entrez *gene ID 74728) and *Ta0871 *that encodes a hypothetical protein from *Thermoplasma acidophilum *(*entrez *gene ID 1456410).

Comparison of our microarray data and comprehensive listing of potential Foxl2 target genes (Additional file 4) to those generated by two other groups of investigators [6,7] has allowed us to generate a subset of Foxl2 targets that have greater potential of being Foxl2 regulated (Additional file 10). Of particular interest are the genes that are common to all three studies: *Bcl11a, Hoxb5, Mrgpre*, and *Ptpn6 *(Tables 1 and 2). The functions of these genes are described below.

*Mrgpre*, along with *Maff *and *Rspo3*, also appear on the qPCR confirmed gene listing of Batista et al. [6]. The *Mrgpre *gene product is a *Mas1 *related G protein coupled receptor that may be involved in the sensation or modulation of pain in a subset of sensory neurons [27]. *Maff *stands for v-maf musculoaponeurotic fibrosarcoma oncogene homolog f (avian). The protein encoded by the gene is a basic-leucine zipper (bZIP) transcription factor that is up-regulated by pro-inflammatory cytokines in myometrial cells [28]. *Rspo3 *encodes R-spondin 3, a secreted protein that mediates *Wnt *signaling and is involved in angiogenesis during mouse development [29].

Finally, the *OKdb *was utilized to identify genes from this study that were demonstrated to be expressed in the ovary in previous studies. These genes have been divided into three tables based on known functions: 1. gene regulation; 2. signaling; and 3. metabolism, cell adhesion, cytoskeletal, and structural. Our focus now turns to a discussion regarding the functions of genes listed in Tables 1, 2, 3 that are known to be expressed in granulosa cells (*OKdb*).

In the area of gene regulation (Table 1), *Bcl11a *and *Serpine2 *are expressed in granulosa cells although their function in the ovary is unknown. The zinc finger transcription factor *Bcl11a *was shown to be up-regulated in human granulosa cells treated with FSH [30]. In human erythroid cells where much more is known about the factor, the *Bcl11A *protein functions as a repressor and is involved in silencing fetal hemoglobin expression in adults [31]. *Serpine2 *is a serine protease inhibitor that is differentially expressed in large and small follicles in sheep [32]. *Serpine2 *protein levels are elevated in dominant bovine follicles [33], whereas the levels of *Serpine2 *are lower in ovaries of Foxl2 knockout mice suggesting that the gene is induced by Foxl2 [7]. *Gabpa *and *Nr4a2 *are two genes in this category that have been characterized to a greater extent with respect to granulosa cell function. *Gabpa *is an ETS family transcription factor that regulates the *Rhox5 *homeobox gene in rat granulosa cells [34]. In the regulation of the nicotinic acetylcholine receptor gene, *Gabpa *recruits the histone acetyl transferase p300 when the promoter is activated, and recruits the histone deacetylase HDAC1 when the promoter is not activated [35]. *Nr4a2 *was found to be rapidly induced by cAMP in the KGN granulosa cell line [36]. LH was shown to induce *Nr4a2 *expression in mouse granulosa cells [37].

Six genes involved in signaling (Table 2) are expressed in granulosa cells. *Akt1 *is a component of the phosphoinositide 3'-OH kinase (PI3K) pathway and is phosphorylated in response to Igf1 stimulation of bovine granulosa cells [38]. *Akt1 *has also found in human granulosa cells during follicle development [39]. The human GnRH receptor has been shown to be expressed predominantly in granulosa cells of pre-ovulatory follicles [40]. The role of GnRH in the ovary is diverse as it regulates steroidogenesis, cell proliferation, and apoptosis [41]. *Gucy1b3 *encodes a guanylate cyclase that is activated by nitric oxide (NO) and is expressed at high levels in granulosa cells of primordial and primary follicles of the rat ovary [42]. NO has been shown to inhibit estrogen production in rat granulosa cells [43] and steroidogenesis in porcine granulosa cells [44]. *Ppp1r1b *is a protein phosphatase involved in signal transduction pathways in human granulosa cells in response to dopamine and human chorionic gonadotropin stimulation [45]. *Prlr *encodes the prolactin receptor which is localized to granulosa cells as well as other cell types in the rat ovary [46]. Prolactin receptor expression in rat granulosa cells is increased by treatment of cultured cells with FSH, LH and hCG [47]. *Ptpn6 *encodes a protein tyrosine phosphatase that is involved in modulating the signaling cascade activated by PRL in granulosa cells [48].

The *Ctla4, Eda*, and *Mrgpre *genes are in the signaling category but are not found in the *OKDB*. However, they are worthy of mention due to appearing in this study as well as both the human KGN study [6] and the mouse knockout study [7]. *Ctla4 *encodes cytotoxic T-lymphocyte associated protein 4, a receptor/signal transducer that suppresses immune system function and is regulated by the forkhead transcription factor FoxP3 [49-51]. Transcriptional regulation of *Ctla4 *by Foxl2 in granulosa cells may be the result of similarities in the Forkhead binding sequence elements in the *Ctla4 *promoter that allow both factors to regulate the gene, with cell type determining the presence of either FoxP3 or Foxl2 in T cells or granulosa cells respectively. The *Eda *gene encodes the protein ectodysplasin A, a tumor necrosis factor family member with several isoforms, one of which is a transmembrane protein [52]. Mutations in the soluble form of the EDA protein and the EDA receptor are the cause of anhidrotic ectodermal dysplasia, a syndrome that results from impaired development of skin appendages during embryogenesis [53].

Genes in Table 3 that have been shown to be expressed in granulosa cells include *Hspg2*, an anticoagulant heparin sulfate proteoglycan involved in follicle development and ovulation in rats[54]. Hspg2 had also been found in human follicular fluid [55]. *Odc1 *encodes ornithine decarboxylase 1 (ODC1), the rate-limiting enzyme of the polyamine biosynthesis pathway which catalyzes ornithine to putrescine. ODC1 expression is stimulated by LH in granulosa cells and may mediate the effects of LH during the process of follicular development [56]. *Thbs2 *encodes thrombospondin 2, an antiangiogenic protein involved in follicle development [26].

Two genes involved in metabolism, *StAR *and *Vldlr*, are expressed in granulosa cells and the gene products of both are involved in steroidogenesis. *StAR *encodes the steroidogenic acute regulatory protein, which transfers cholesterol from the outer to the inner mitochondrial membrane, the rate limiting step in steroidogenesis [57]. *Vldlr *encodes the very low density lipoprotein receptor, which obtains lipoproteins from plasma, a source of cholesterol for steroidogenesis [58].

### Transient co-transfection and luciferase assays

Foxl2 over-expression resulted in a 6 fold activation of the GnRHR gene promoter in transient co-transfections of KK1 cells. This was the first demonstration of Foxl2 regulation of the GnRHR gene in ovarian derived cells. Our previous study had demonstrated that Foxl2 could potentially regulate the GnRHR promoter in pituitary derived αT3-1 cell line based on activation by the Foxl2-VP16 fusion protein [9]. Foxl2-VP16 action was directed by binding to the GRAS element with the potential for complex formation with Smad and AP1 transcription factors [9]. Whether a similar mechanism or alternative binding site(s) are involved in granulosa cells has not been determined.

Only a few Foxl2 target genes in the ovary have been confirmed through promoter cloning and reporter gene fusion analysis. The goat *CYP19 *gene, which encodes the enzyme aromatase, was activated by Foxl2 [59]. The human *StAR *gene, encoding the steroidogenic acute regulatory protein, is repressed by FoxL2 [15]. We have evidence that the mouse *StAR *gene is also repressed by Foxl2 (in preparation).

## Conclusions

We have identified potential Foxl2 regulated ovarian genes through microarray analysis and comparison of our data to that from other microarray studies. Foxl2 derivatives with either activation or repression domains were used in this gene discovery process. Foxl2 regulation of steroidogenesis appears to be of importance in the ovary, as many of the genes we identified appear to be involved either directly or indirectly in the process. These include *GnRHR*, *Gucy1b3, Prlr, Ptpn6, StAR *and *Vldlr*.

The *GnRHR *gene identified through microarray analysis has been validated as Foxl2 responsive through promoter cloning and reporter gene analysis. Transient co-transfections of a GnRHR-luciferase reporter vector and a wild-type Foxl2 expression vector provided evidence of Foxl2 transcriptional activation of the GnRHR gene promoter in the mouse ovary derived KK1 granulosa cell line.

## List of abbreviations

cAMP: cyclic adenosine monophophate; GnRHR: gonadotropin releasing hormone receptor; StAR: steroidogenic acute regulatory protein; BPES: blepharophimosis/ptosis/epicanthus inversus syndrome; POF: premature ovarian failure; LacZ: β-galactosidase; DMEM: Dulbecco's Modified Eagle's Medium; F12: Ham's F12 nutrient mixture; FBS: fetal bovine serum; SID: mSin3 interaction domain; μg: microgram; μl: microliter; *OKdb: Ovarian Kaleidoscope Database; *FSH: follicle stimulating hormone; NO: nitric oxide; LH: luteinizing hormone; hCG: human chorionic gonadotropin

## Competing interests

The authors declare that they have no competing interests.

## Authors' contributions

JME designed and performed the transient transfection studies and helped develop the manuscript. JLH performed the microarray data analysis and revised the manuscript.

CMC cloned the murine GnRHR promoter, cloned the murine Foxl2 gene, and revised the manuscript. KWE designed and performed the microarray study, performed data analysis, and developed the manuscript. All authors have read and approved the final manuscript.

## Supplementary Material

Additional file 1 **Pairwise analysis of differentially expressed genes in KK1 cells**. Group NA: mock vs. VP16. Click here for file

Additional file 2 **Pairwise analysis of differentially expressed genes in KK1 cells**. Group NR: mock vs. Mad. Click here for file

Additional file 3 **Pairwise analysis of differentially expressed genes in KK1 cells**. Group RA: Mad vs. VP16. Click here for file

Additional file 4 **Comprehensive listing of potential Foxl2 target genes of known function generated by microarray analysis**. Group designations: (-) decreased fold expression; (+) increased fold expression. NA = Normal: Activated (mock transfected compared to Foxl2-VP16 transfected). NR = Normal: Repressed (mock transfected compared Foxl2-Mad transfected). RA = Repressed: Activated (Foxl2-Mad compared to Foxl2-VP16). E16 = mouse embryonic stage day 16. E13 = mouse embryonic stage day 13. P0 = mouse birth stage. Click here for file

Additional file 5 **Combined analysis of differentially expressed genes in KK1 cells**. Normal: mock transfected. Activated: Foxl2-VP16 transfected. Repressed: Fodl2-Mad transfected. Click here for file

Additional file 6 **Analysis of differentially expressed genes in E13**. Wild type vs. Foxl2 knockout. Click here for file

Additional file 7 **Analysis of differentially expressed genes in E16**. Wild type vs. Foxl2 knockout. Click here for file

Additional file 8 **Analysis of differentially expressed genes in P0**. Wild type vs. Foxl2 knockout. Click here for file

Additional file 9 **Comparison of differentially expressed genes in KK1 cells to E13, E16, and P0**. Sheet 1 lists 225 genes common to KK1 and E13. Sheet 2 lists 211 genes common to KK1 and E16. Sheet 3 lists 325 genes common to KK1 and P0. Sheet 4 lists 5 genes common to all 3 groups. Sheet 5 lists 22 common genes. Sheet 6 lists 29 common genes. Sheet 7 lists 21 common genes. Sheet 8 lists 155 genes unique to KK1 & E16. Sheet 9 lists 177 genes unique to KK1 & E13. Sheet 10 lists 270 genes unique to KK1 & P0. Click here for file

Additional file 10 **Potential Foxl2 target genes validated by comparison to data from other microarray studies**. Gene *Functions *were obtained from the *Ovarian kaleidoscope data base*^1^, NCBI RefSeq^2^, and UniProtKB/Swiss-Prot^3^. All genes listed that do not have an asterisk after the gene symbol are common to our study and the *in vivo *in mouse ovary study only [7]. Genes denoted by a single asterisk* are common to our study and the human KGN cell line only [6]. Those indicated by ** are common to all three studies. The StAR*** and GnRHR*** genes were found to be Foxl2 regulated in our study and do not appear in the KGN [6] or *in vivo *[7] studies. Fold change numbers indicate relative gene expression levels. Group designations: (-) decreased fold expression; (+) increased fold expression. NA = Normal: Activated (mock transfected compared to Foxl2-VP16 transfected). NR = Normal: Repressed (mock transfected compared Foxl2-Mad transfected). RA = Repressed: Activated (Foxl2-Mad compared to Foxl2-VP16). E16 = mouse embryonic stage day 16. E13 = mouse embryonic stage day 13. P0 = mouse birth stage. Click here for file
